# Parental responses to children’s early health disadvantages: evidence from a British twin study

**DOI:** 10.1093/esr/jcae016

**Published:** 2024-03-18

**Authors:** Alicia García-Sierra

**Affiliations:** Swiss Centre of Expertise in Life Course Research LIVES, Université de Lausanne, Bâtiment Géopolis, 1015 Lausanne, Switzerland

## Abstract

Health problems experienced in the early years of life have detrimental consequences for the entire life course. However, parents can, through their child-rearing actions, alleviate or aggravate these effects. This article examines how parents respond to the early physical health disadvantages suffered by their children and whether parents from high- and low-socioeconomic backgrounds develop different responses to their children’s early health problems. Using longitudinal data from the Twins Early Development Study, I implement a series of within-twin fixed-effects models and find that, on average, parents develop more negative emotional responses and implement harsher discipline behaviours when their children experience an early health problem. Surprisingly, the effect of health problems on parental responses does not differ by the socioeconomic status of the family. With some nuances, this evidence suggests that parental responses reinforce early-in-life health disadvantages.

## Introduction

The consequences of a health problem experienced in early life may, in many instances, extend into adulthood. This is why both the short-run (Currie, 2009) and the long-term effects (Smith, 2009) of early health problems have been extensively explored. This relationship between early disadvantages and later-in-life outcomes operates through two channels (Yi *et al.*, 2015). First, through a direct or biological pathway whereby health problems experienced in the first years of life might persist until later ages and increase the likelihood of developing other conditions. Second, studies reveal an indirect channel by which the effect of children’s early human capital on later-life outcomes may be influenced by how parents respond to these early disadvantages (Currie and Almond, 2011).

In this article, I focus on this indirect pathway and examine how parents respond to early health problems suffered by their children. Although there is a growing literature exploring the formation and impact of parental responses on their children’s lives, it is still unclear the specific role that parents play when the disadvantage in question is health-related (Rosales-Rueda, 2014; Yi *et al.*, 2015). This study aims to fill this gap by shedding light on the determinants of parental responses to early physical health problems in England and Wales.

Besides, and differently from most of the previous literature, which focuses on monetary investments as responses to early health disadvantages (Yi *et al.*, 2015; Savelyev *et al.*, 2020), I examine parents’ emotional and discipline-related responses. These two types of parental responses are crucial because (i) they have been noted to get triggered by children’s related stressors (Greenwald *et al.*, 1997; Barnett *et al.*, 2003; Leerkes and Augustine, 2019); and (ii) they impact the process of child development (Knafo and Plomin, 2006; Viding *et al.*, 2009; Tocu, 2014).

Parental emotional responses are conceptualized in this article as the feelings of anger, frustration, impatience, or non-attachment that parents develop elicited by their children. Parental discipline responses are understood as a series of behaviours such as smacking, telling off, making a joke out of the children’s misbehaviour, asking someone else to deal with a situation related to the children, avoiding explaining or reasoning with the children, and being lax with them.

This article is organized around two interrelated questions. First, I study *how parents respond to the early health disadvantages suffered by their children.* Second, following the existing evidence showing that more socioeconomically advantaged parents are more likely to positively respond to other types of early problems suffered by their children (Hsin, 2012; Restrepo, 2016; Torche, 2018), I test whether *parents from high- and low-socioeconomic backgrounds respond differently to their children’s early health problems*.

Using data from the Twins Early Development Study (hereafter TEDS), a longitudinal database following twins in England and Wales for more than 20 years, I implement a series of within-twins fixed-effects models. This allows me to measure the effect that early physical health problems have on parental emotional and discipline responses later in life. I measure early health disadvantages during the first 18 months of life of the twin through the Twin Medical Risk Scale and parental responses at three different points of the childhood period (ages 3, 4, and 7) in the emotional and discipline dimensions.

The findings suggest that parents respond negatively to their children experiencing early health problems, both by developing negative emotional responses and by implementing harsher discipline behaviours. This suggests that parents reinforce existing disadvantages within the family. Surprisingly, there are no socioeconomic differences in how parents respond to early health problems, whether socioeconomic status is measured as parental education, occupation, or household income.

This article adds to the existing literature in several ways. First, it makes a substantive contribution by delving into the effect of early health problems on two previously unexplored parental responses, namely, emotional and discipline-related behaviours. Second, it expands previous methodological approaches to the study of parental responses employing twins’ fixed effects by adding a longitudinal design with repeated measures of parental responses. Third, by exploring the UK case, this article adds to the existing research exclusively focused on the United States and China, where public health systems are much more limited in their scope. Finally, this paper contributes to the previous literature exploring parental responses to health disadvantages by opening the door to heterogeneous SES effects, which has been noted to be a crucial dimension to understanding parental responses to other types of early child disadvantages.

## Theoretical background

### Parental responses to early disadvantages

Early health problems have a long-lasting impact on children’s cognitive development, later health, or educational attainment (Currie and Almond, 2011). As Yi *et al.* (2015) acknowledged, early health problems do not only operate through a direct biological channel but also through an indirect pathway in which parental responses play an important role. Therefore, how parents respond to these health disadvantages can be crucial to mitigating or amplifying the effect of these early problems on later outcomes.

Previous literature exploring parental responses to children’s endowments (i.e., cognitive skills, developmental milestones, or birth weight) has found mixed evidence of the existence of reinforcing and compensatory responses. A reinforcing response means that parents (consciously or unconsciously) reduce their investments in their children when they show some developmental problems, which eventually reinforces the original difficulty (Datar, Kilburn and Loughran, 2010; Frijters *et al.*, 2013). In the case of compensatory responses, parents increase the investments in the aggrieved child in an attempt to compensate for that initial handicap (Halla and Zweimüller, 2014; Fan and Porter, 2020). The ambiguity in these results can be explained by the type of endowment and parental response considered (Ayalew, 2005; Yi *et al.*, 2015), the socioeconomic status of the family (Restrepo, 2016), or the type of population explored (i.e., low- vs. high-income countries) (Savelyev *et al.*, 2020).

When considering parental responses to health problems, which is an essential dimension of human capital, the previous results also show three possible scenarios: reinforcement (Ayalew, 2005), compensation (Bharadwaj, Eberhard and Neilson, 2017; Terskaya, 2019; Savelyev *et al.*, 2020), or mixed results. Among the last ones, Rosales-Rueda (2014), using US data, does not find differences in the parental responses when the children suffer a physical health problem, but finds evidence of reinforcing parental behaviour when children experience mental health problems. Also, Yi *et al.* (2015) use Chinese data and a twins’ design and show that the sick twin receives more health investments but fewer educational resources than the healthier twin.

The vast majority of these studies come from the field of health economics and present two common limitations. First, they examine an essentially economic outcome, namely, parental monetary investments, as the main form of parental responses (Yi *et al.*, 2015; Savelyev *et al.*, 2020). However, from the broader sociological literature, it is well known that parental responses can take multiple forms such as cognitive stimulating behaviours (Price and Kalil, 2019), parent–children quality interactions (Grätz and Torche, 2016), or parental closeness (Kalil, Ryan and Corey, 2012), all of them very relevant to explain children’s development and life trajectories (Smeeding, Erikson and Jäntti, 2011; Schiffrin and Liss, 2017; Bornstein, Putnick and Suwalsky, 2018).

The second limitation of these studies is that they usually work under the assumption that parenting is a rational process and that parents will base their investment decisions on the costs and benefits (Becker and Tomes, 1976). Recent sociological approaches such as Kalil (2015) question this paradigm and suggest that parents cannot fully decide their parenting based on costs and benefits because these are only visible years after the investments have been made. Therefore, a large part of the sociological research on this topic has argued that there is not such a thing as a universal logic of parenting driving all the parental responses to children’s disadvantages, and evidence of this is that high- and low-SES families usually present different parenting approaches. According to this argument, more disadvantaged parents are less likely to implement what the literature considers an effective parental behaviour simply because they are unaware of the effectiveness of certain behaviours for their children’s development (Conley, 2008; Rowe, 2008).

In this article, I focus on the effect of early health disadvantages on parental emotional responses and discipline-related behaviours, which differ in their nature from monetary investments and which have been noted in the sociological and psychological theory to be crucial elements of the parent–child interaction dynamics (Larsson *et al.*, 2008; Glover *et al.*, 2010; Mackenbach *et al.*, 2014).

### Parental negative emotional responses

As defined by Leerkes and Augustine (2019: p. 620), ‘parenting is an inherently emotional task’. That is why, children’s characteristics and behaviours might elicit certain feelings among their parents that are conceptualized as parental emotions. In this article, I focus particularly on negative emotional responses, such as experiencing anger, frustration, impatience, or non-attachment.

Several children’s characteristics have been shown in the literature to trigger negative parental emotional responses. Kiff, Lengua, and Zalewski (2011) find that child temperament generates negative parental emotional responses and Mullineaux *et al.* (2009) find that parents exhibit more negative emotions during middle childhood when the child has experienced more conduct problems in the early years of life. Finally, Stewart *et al.* (2017) prove that child developmental disorders produce negative emotions and stress in their parents.

As suggested in Leerkes and Augustine (2019), the process by which these negative emotional responses are produced follows the next steps. First, a stressor associated to the child emerges. Second, parental empathy is activated through a complex neurobiological process. Third, this triggers the emergence of negative emotions such as anger or frustration. Fourth, in some cases, parents can regulate these emotions to adapt them to the children’s situations, although this will depend on the parental-specific abilities.

According to existing research, one of the most important stressors related to children is health. As pointed out by Barnett *et al.* (2003: p. 185) it is well known that children’s medical problems foster ‘unique emotional and physical demands that stress and strain parents’. Those parents whose children go through an illness deal with high levels of uncertainty, frequent medical visits, and caring workload. This all has an impact on parents’ mental health, being responsible for high levels of stress (Teti, O’Connell and Reiner, 1996).

Since stress is highly associated with negative emotions, children’s health problems might activate negative emotional responses from parents (Leerkes and Augustine, 2019). Therefore, the first hypothesis of this article suggests that (**hypothesis 1a**) *parents will develop negative emotional responses when their children experience early health problems.*

The relevance of parental emotional responses comes from the fact that exposure of the children to the negative emotional states of the parents affects parental behaviour and, eventually, children’s outcomes (Kurtz-Nelson and McIntyre, 2017). For instance, negative parental emotions are associated with more restrictive and harsh parental behaviours (Leerkes and Augustine, 2019), and parenting-related anger has been shown to be conducive to physical punishment and authoritarian parenting (Bryan and Dix, 2009).

Regarding children’s outcomes, Glover *et al.* (2010) show that parents’ negative emotional responses positively correlate with children internalizing and externalizing behaviours during the early teenage period. Additionally, Larsson *et al.* (2008) give evidence of the association between parental negative emotional responses and children’s conduct problems. Not only are these emotional responses associated with behavioural problems but also with learning abilities and the cognitive developmental process of the children (Park *et al.*, 2005).

### Parental negative discipline responses

Parental discipline strategies are also considered a central component of the socioemotional interactions that parents and children hold (Laskey and Cartwright-Hatton, 2009). The term parental discipline usually encompasses two different types of behaviours: (i) a corporal one which includes physical punishment like spanking that causes pain for the children; and (ii) a non-physical one which usually includes ‘time-outs’ or penalising the child in different ways (Ryan *et al.*, 2016).

Although there have been attempts to reframe the concept towards a more positive or effective understanding of the term, the most common terminology (and the one used here) still refers to maladaptive parental discipline (Grusec *et al.*, 2017). The conceptualization used in this article encompasses the following behaviours: smacking, telling off, making a joke out of the children’s misbehaviour, asking someone else to deal with a situation with the child, avoiding explaining or reasoning with the children, and being lax with them.

Among the determinants for parental discipline, some studies point out the relevance of children’s misconduct or non-compliance, personal distress (Masarik and Conger, 2017), or family problems (Rodriguez, 2016). Similarly to the emotional response case, children-related stress has been shown to increase the likelihood of implementing harsh discipline behaviours (Fisher, 1991).

Health problems might work as a stressor that increases parental irritability, which is a key predictor of disciplined parenting (Greenwald *et al.*, 1997). Moreover, negative emotions, when not appropriately regulated (Lunkenheimer *et al.*, 2023), also foster harsher parental discipline (Lorber, 2012), so this could also be an indirect channel through which children’s health impacts parental harsh discipline behaviour.

Therefore, I suggest that (**hypothesis 1b**) *parents will respond to the early health problems of their children by implementing harsh discipline behaviours.*

Understanding what determines negative parental discipline is important because, in the long term, punitive parental discipline impacts the sense of autonomy of the child, as well as the security and availability of parental support (Laskey and Cartwright-Hatton, 2009). This lack of parental support has been shown to impact children’s self-esteem and educational performance in the long term (Wong, 2008), as well as their developmental outcomes (Mackenbach *et al.*, 2014).

### Heterogeneous SES responses

The compensatory advantage literature (Bernardi, 2014; Bernardi and Grätz, 2015; Bernardi and Triventi, 2020) has suggested that exploring the average parental responses to children’s disadvantages might camouflage inequalities across families from different socioeconomic positions. For instance, high-SES parents are more likely to compensate with their actions and resources for the negative events experienced by their children than low-SES parents (Torche, 2018). Therefore, children from socioeconomically advantaged contexts are less dependent on prior negative outcomes, as compared to those from disadvantaged family contexts (Restrepo, 2016). A similar argument has been made by the literature on the stratified logics of parenting (Baier, 2019), which relies on Lareau’s (2011) work and differentiates between the parental strategies and actions of socioeconomically advantaged and disadvantaged parents.

Yet, it remains mostly unknown whether these differences in parenting are in place in the case of parental responses to early health problems. There are several specific health-related reasons to think that parental socioeconomic background might condition parental responses to children’s health problems.

First, Cunha (2015) shows that high-SES parents are more likely to have more parenting knowledge, as well as have more resources to pay for private health care, which implies that they are in more contact with professionals and are more likely to receive anticipatory guidance from healthcare institutions. However, low-SES parents are more prone to turn to their extended family or community networks, which usually implies less updated and rigorous information flows (Berkule-Silberman *et al.*, 2010). This is important because holding more accurate knowledge about child development has been linked to more realistic expectations, less frustration and anger, and, in general, less negative emotional responses and harsh discipline behaviours (Leerkes and Augustine, 2019).

Second, applying the overall logic of the family stress model paradigm (Masarik and Conger, 2017), it is also reasonable to expect higher levels of susceptibility to health problems in low-SES contexts. In these environments, parental self-efficacy is usually lower (Whitbeck *et al.*, 1997), which also increases the likelihood of experiencing irritability, and triggers negative parental emotional responses such as anger, non-attachment, impatience or frustration (Ryan *et al.*, 2016), and particularly, harsh discipline behaviours (Conger, Conger and Martin, 2010; Masarik and Conger, 2017).

Following all this, I expect that (**hypothesis 2a**) *low-SES parents will develop more negative emotional responses* and (**hypothesis 2b**) *implement more harsh discipline behaviours as a response to early health problems than high-SES parents.*

## Data and methods

### Data

The data used for this article comes from the Twin Early Development Study (TEDS), a longitudinal data collection that follows more than 10,000 pairs of twins born in England and Wales between 1994 and 1996 during their childhood, adolescence, and early adulthood (Rimfeld *et al.*, 2019). Information on twins’ health, parental responses, and long-term outcomes is available across six waves from age 1 to age 21.

There are three main advantages of this dataset. First, the longitudinal character of the data allows me to capture the health status of the children at a very early age, as well as the parental responses several times during the childhood period up until early adulthood. Secondly, it follows an extended twin family structure, meaning that it contains individual information about each of the two twins and their parents. Finally, the variables presented in the data combine self-reported measures (provided by the twins themselves) with parental reports, which increases their reliability.

Individual twins nested in families are the main units of observation. The sample size varies across waves, with 25,801 twins with health records at age 1, 11,595 individuals with parental response information at age 3, 15,295 at age 4, and 14,745 at age 7. Explorations of attrition patterns show that the experience of health problems during childhood does not predict the likelihood of a family unit leaving the sample.

Moreover, it is important to understand this study in the context of the United Kingdom, where the National Health System offers a comprehensive service to the citizens, and therefore, it might be easier for parents to detect early health problems than in other contexts without universal health care.

### Variables

The independent variable of this study is the Twin Medical Risk Factors Scale, a twin-specific health composite index measured during the first 18 months of life of the twin, originally developed by Pike *et al.* (2006) and later introduced as part of the core TEDS-derived variables. This variable aims to capture the underlying health status of the twins by including four factors that increase the vulnerability of the children during their first years of life: (i) days in special care, (ii) medical problems at birth, (iii) low birth weight, and (iv) days in the hospital after birth.

The first item contributing to the composite, days in special care, captures the total number of days that the child has spent in the neonatal care area as well as the paediatric special care units during the first year and a half of life, with a minimum of 0 days and a maximum of 100 days. The second item is a binary indicator that measures whether the twin has experienced medical problems at birth. The low-birth-weight variable (reversed) measures the weight of the twin at birth in grams, ranging from 400 to 4500. Finally, the days in hospital variable measures the days the twin stayed in the hospital after birth, with a minimum value of 0 days and a maximum of 60. The four items were originally selected through a process of exploratory factor analysis,^1^ and the level of internal consistency is 0.87 (Cronbach Alpha).

The process of construction of the variable by the TEDS data centre is the following: (i) twins with unknown zygosity or gender information, as well as those who experienced very severe perinatal problems are excluded, (ii) the four items contributing to the twin medical risk scale are standardized, and outliers are removed, (iii) the standardized version of the four variables are summed, and (iv) the final composite for each twin is standardized obtaining a scale ranging from −3 to 6 with mean 0 and standard deviation 1. The individuals with higher values in this variable are those with a worse health status (i.e., larger medical risk).

This composite measure has several advantages when compared to other health measures or proxies used in the literature. The most popular alternative would be to use an indicator of birth weight exclusively, which has been extensively used. However, there are three good reasons to opt for the twins’ medical risk composite instead of the birth weight measure. First, when exploring parental responses, it is important to consider that more explicit health problems will be more easily identified by the parents. Second, birth weight is generally low among twins, given their particular gestational process, and therefore, it does not necessarily reflect a particularly worse health status on its own (Gielen *et al.*, 2007). Third, whereas birth weight is measured at an isolated point in time, the medical risk composite is extended during the first 18 months of the life of the children.

Finally, it is relevant to notice that the Twin Medical Risk Factor Scale has been previously used in the literature exploring early developmental processes (see Asbury, Wachs and Plomin, 2005 for an example) and it has been found to increase the risk of developing other health conditions during their later childhood and adult life (Martin *et al.*, 2017; and see Koeppen-Schomerus *et al.*, 2000; Ronald *et al.*, 2010 for examples using the TEDS sample).

The main dependent variable is child-specific parental responses, operationalized with two indicators: negative emotional responses and discipline behaviours.

First, I measure negative parental emotional responses towards the children at ages 3, 4, and 7 through a derived composite index offered as part of the TEDS core dataset. Parents are asked to rate from 1 (definitely untrue) to 5 (definitely true) if sometimes they feel (i) angry, (ii) frustrated, (iii) impatient, or (iv) non-attached to the twins. The answers to the four items are standardized, and the mean is computed for each twin.^2^ The final index presents a mean of 0 and a standard deviation of 1, ranging from −6 to 6. Higher values capture stronger negative emotional responses. At age 3 the four items present an internal consistency of alpha 0.83, at age 4 of 0.84, and at age 7 of 0.80 (in line with Larsson *et al.*, 2008).

During the survey, parents are asked to provide the answers for the first-born twin, and immediately afterwards, they are asked if they feel more or less that way with the second-born twin, to what they can choose from 1 (a lot more) to 5 (a lot less). This implies that parents do necessarily focus on the potential differences between the two children (Viding *et al.*, 2009). This is an important advantage for the methodological design of this study, which exploits within-twins variation. The Intraclass Correlation Coefficient^3^ (ICC hereafter) is 0.61 at age 3, 0.60 at age 4, and 0.55 at age 7, which suggests that there is enough variation for this study to exploit within-family differences.

Before being incorporated as part of the TEDS survey, this composite was originally developed by Deater-Deckard (2000) through a process of exploratory factor analysis to measure the relevance of parental emotional responses for the child developmental process, and it has been extensively used in the parenting literature since then, becoming the standardized way of measuring parental emotional responses (Asbury, Dunn and Plomin, 2006; Larsson *et al.*, 2008; Viding *et al.*, 2009; Leerkes and Augustine, 2019).

The second operationalization of parental responses is through a composite measuring parental discipline at ages 3, 4, and 7. Parents are asked how often they implement these actions with each of the twins (from never to usually): (i) giving a smack or slap, (ii) telling off or shouting at the twin, (iii) making a joke out of the children’s misbehaviour, (iv) asking someone else to deal with the situation (for example, another parent), (v) explaining to the child, or reason with the child, and (vi) being firm and calm with child. The first two dimensions, giving a smack or slap, constitute harsh discipline behaviours. The second pair, making a joke or asking someone else to deal with the situation, are considered displacement behaviours in the literature (Knafo and Plomin, 2006). Finally, the third pair, reasoning with the child and being calm are understood as positive discipline behaviours. Because of this, and after having computed the mean of each subscale and standardized them, the first and third subscales are summed and the second one is subtracted. The final composite presents a mean of 0 and a standard deviation of 1, ranging from −6 to 6, with higher values capturing more negative parental discipline. The internal consistency of the six standardized measures included in this composite is 0.79 (Cronbach alpha) at age 3, 0.82 at age 4, and 0.80 at age 7.

As in the previous composite, parents are also asked first about the first-born twin and then to capture the differential treatment between twins and how that compares to the second-born twin. This again ensures enough variations within the family in the levels of parental discipline, with ICCs of 0.67 at age 3, 0.68 at age 4, and 0.55 at age 7.

This composite has been extensively used in the literature exploring parental discipline (Locke and Prinz, 2002; Choe, Olson and Sameroff, 2013; Lysenko, Barker and Jaffee, 2013), especially using TEDS data (Asbury, Dunn and Plomin, 2006; Knafo and Plomin, 2006; Fontaine *et al.*, 2010; Oliver, Trzaskowski and Plomin, 2013).

In the second stage of the analyses, where I explore heterogeneous SES effects, I operationalize family socioeconomic background through a dominant version of parental education (i.e., I account for the highest qualification attained by the mother or father in the family). This is a dichotomous variable with a value of 0 if both parents have achieved less than tertiary education and 1 if any of the parents have achieved a tertiary education degree. I replicate the main analyses in the sensitivity analyses section using a more disaggregated operationalization of parental education with six categories, as well as a measure of mothers’ occupations and household income.

Given the characteristics of the within-twins estimators, I can only include twin-specific control variables in the main analyses, which is the case of twins’ gender. In the OLS specifications, I also control for relevant factors that vary between families, such as parental education, the age of the mother when the children were born, and the number of siblings in the family (including the twins). Further sensitivity tests include controls for the cognitive ability of the twin at age 3, the zygosity of the twins’ pair, and the gender of the parent responding to the survey. Descriptive statistics of all the variables are presented in Supplementary Appendix Table A1.

### Analytical strategy

Health problems do not randomly occur across children and families, which creates a problem of selection because there might be unobservable characteristics that are related to both the parental responses and the health problems of the children (Rosales-Rueda, 2014). Examples of these unobservable factors would be housing conditions, neighbourhood-related factors, or health problems experienced by the parents themselves. Therefore, to overcome this selection problem, I exploit within-family variation, which allows me to also control for observed and unobserved family characteristics. Another advantage of this specific design is that, as compared to siblings’ models, twins share parental age at birth and the timing of most of their early life events (Abufhele, Behrman and Bravo, 2017).

The analytical strategy of this article has two interrelated parts. The first stage aims to capture the effect of early health disadvantages on parental responses (*hypotheses 1a* and *1b*). First, I present an OLS regression to have a baseline estimate of the relationship between the variables of interest, followed by a series of within-twins fixed effects linear models, which are the central part of the analyses. These twin-fixed effects models compare twins living in the same household. Formally, the resulting model for this first stage of the analysis is:


$$\begin{array}{lll} {Y_{p}}_{t}-{\bar{Y}}_{p}= & \;\delta \;\left({X_{p}}_{t}-{\bar{X}}_{p}\right)\; & +\theta \left({C_{p}}_{t}-{\bar{C}}_{p}\right)+\left({E_{p}}_{t}-{\bar{E}}_{p}\right), \end{array}$$ (1)

where Y is the main outcome, parental responses; subscript p refers to the twins-pair and t  to the individual-twin; X refers to the children’s early health disadvantages, the main predictor, and *C* to the control variables. The main estimate of interest is δ.

The second stage of the analysis focuses on whether the effect of early health disadvantages on parental responses varies by the socioeconomic background of the family, as suggested in *hypotheses 2a* and *2b*. Equation (2) introduces an interaction term between early health problems (X) and parental education (S):^4^


$$\begin{array}{lll} {Y_{p}}_{t}-{\bar{Y}}_{p}= & \;\delta \left({X_{p}}_{t}-{\bar{X}}_{p}\right)+\;\;\omega \left({X_{p}}_{t}-{\bar{X}}_{p}\right)*S_{p}\; & +\theta \left({C_{p}}_{t}-{\bar{C}}_{p}\right)+\left({E_{p}}_{t}-{\bar{E}}_{p}\right),\;\;\;\;\;\;\;\;\;\;\; \end{array}$$ (2)

where, as in Equation (1), Y is the main outcome, parental responses; X captures children’s early health disadvantages, and S the socioeconomic status of the family. The main estimate of interest in this case is ω.

All the analyses have been performed with R Studio (package *plm*). A detailed discussion of how this analytical strategy addresses potential sources of bias can be found in the Supplementary Materials.

## Results

### Descriptives

The within-family estimators measure the deviation of each twin-exclusive characteristic from the specific mean of the twin pair. Thus, the underlying assumption of the model is that the twins should present variation in their health status. The Twin Medical Risk Scale, the main independent variable, presents an ICC of 0.63 in this sample.

**Figure 1:**
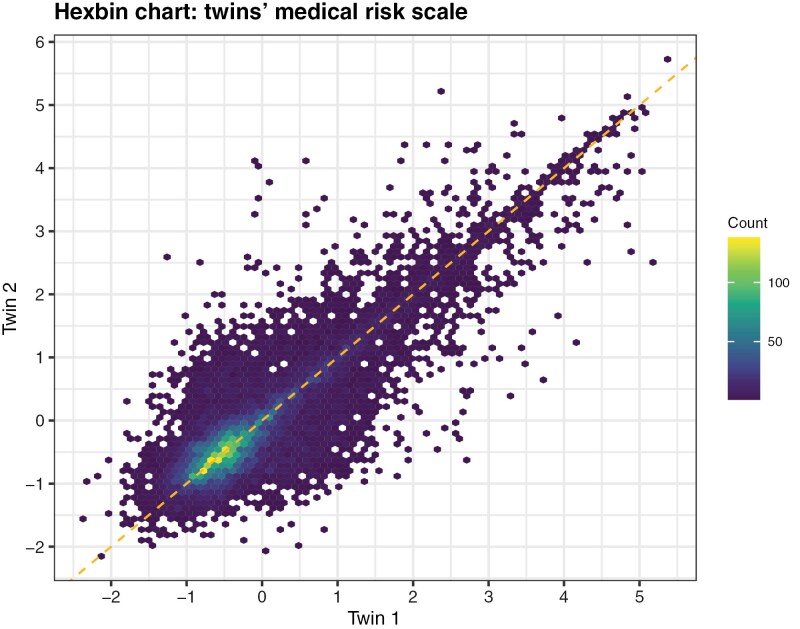
Hexbin chart: twins’ medical risk scale

On top of the ICCs, it is important to look at the within-family variation across different points of the distribution of the medical risk scale. As shown in Figure 1, although there is an evident correlation between the twins’ health status, all the hexagons located out of the main diagonal provide enough variation to sustain this analysis. Even if the highest count of cases falls into the ‘−0.5 −0.5’ coordinates (lightest area) there is a large number of cases for which one twin presents values comprised between 0 and 1 (slightly unhealthy) and the other between −1 and 0 (healthy). The exact differences between the twins’ medical risk scale are the following: 13 per cent of the twins-pairs in the sample have exactly the same medical risk value, 50 per cent of the twins-pairs show differences above 0.3 points (1/3 of the standard deviation), and 20 per cent of the pairs present differences larger than 1 standard deviation.

Both parental emotional responses and discipline behaviours are more similar when the twins are younger, and they become more distinguishable once the twin gets older. There are no differences between SES groups in the level of twins’ similarity: for all the variables employed, the ICCs across groups overlap.

The health status of the twins included in this sample is similar to that of the whole population of British twins. Regarding birth weight, one of the four factors of the twin medical risk index, the mean weight of the TEDS twins is 2,740 grams, close to the 2,500 grams reported by the UK National Twin Statistics (Office for National Statistics, 2006). Although there are no official statistics available for the other factors included in the medical risk composite, the median number of days requiring some type of special care after birth in the sample (14 days), the median number of days hospitalized (7 days), and the probability of reporting medical problems at birth (9.87 per cent of the sample), are in line with previous studies examining special needs (Murray *et al.*, 2020), hospitalizations (Henderson *et al.*, 2004), or severe health problems (Kilby, Gibson and Ville, 2019) among twins in the United Kingdom.

### Twins-fixed effects regressions

Table 1 presents the main results of this study. When family unobserved characteristics are accounted for, having a worse health status (i.e., higher values in the twin medical risk scale) increases the negative emotional responses of parents at ages 3, 4, and 7. As compared to the healthier twin, the twin with worse health during the first year and half of life will receive 0.046 SDs more negative emotional responses from their parents at age 3. This effect becomes more intense in the consecutive years (0.061 and 0.055 SDs at ages 4 and 7), showing similar sizes to the effects found by Rosales-Rueda (2014) for parental emotional and cognitive support (i.e., 0.08 SD). These estimates are totally in line with the results presented in the OLS specification (Supplementary Appendix Table A2), although slightly larger, which suggests that the between-family comparison might to be underestimating the effects of early health problems on parental responses.

**Table 1:** Twins fixed effects models

	Dependent variable					
	Negative emotional responses			Negative discipline behaviours		
	Age 3	Age 4	Age 7	Age 3	Age 4	Age 7
	(1)	(2)	(3)	(4)	(5)	(6)
Twin Medical Risk Scale	0.046^**^ (0.020)	0.061^***^ (0.018)	0.055^**^ (0.019)	−0.011 (0.018)	0.034^**^ (0.015)	0.042^**^ (0.019)
Male	0.161^***^ (0.020)	0.155^***^ (0.018)	0.149^***^ (0.019)	0.249^***^ (0.018)	0.241^***^ (0.015)	0.310^***^ (0.019)
Observations	11,525	15,295	14,745	11,525	15,295	14,745

*Note:* Standard errors are in parentheses.

*** = 0.001, ** = 0.01, * = 0.05.

In the case of parental discipline responses, the pattern is similar: parents implement harsher discipline behaviours with the twin with worse health status. The exception is discipline responses at age 3, which remain unaffected by the twin medical risk scale. This is also the case in the between-families model and suggests that parental responses might be shaped at different rates, namely whereas the emotional responses are already activated at age 3, the behavioural ones seem to take some more time. Finally, it is noticeable and remains constant across all the model specifications that the male twin will receive more negative emotional responses and discipline behaviours from their parents in all cases.

### Heterogeneous SES responses

The second stage of the analysis aims to examine whether parents from high- and low-socioeconomic status respond differently to the early health disadvantages of their children. Table 2 shows that there are no consistent differences across groups in their emotional and discipline responses to health problems. Even though tertiary-educated parents are slightly more likely to develop negative responses than lower-educated ones at ages 3 (model 1) and 4 (model 5), this pattern is not constant.

**Table 2:** Parental responses by family SES

	*Dependent variable*					
	Negative emotional responses			Negative discipline behaviours		
	Age 3	Age 4	Age 7	Age 3	Age 4	Age 7
	(1)	(2)	(3)	(4)	(5)	(6)
Twin Medical Risk Scale (ref. category: Non-Tertiary-Educated)	0.013 (0.026)	0.053^*^ (0.023)	0.059^*^ (0.025)	−0.029 (0.023)	0.005 (0.020)	0.061^*^ (0.025)
Twin Medical Risk × Tertiary-Educated	0.107^*^ (0.047)	0.046 (0.040)	−0.020 (0.043)	0.043 (0.041)	0.081^*^ (0.035)	−0.075 (0.043)
Tertiary-Educated Coefficients^a^	0.121^**^ (0.040)	0.100^**^ (0.034)	0.041 (0.036)	0.017 (0.036)	0.086^**^ (0.031)	−0.012 (0.036)
Controls	Yes	Yes	Yes	Yes	Yes	Yes
Observations	10,240	13,678	13,198	10,240	13,678	13,198

^a^The tertiary-educated coefficients are computed by adding the reference category coefficients to the interaction term ones. The standard errors for these coefficients are calculated in the stratified sample (i.e., rerunning the models only for those with tertiary-educated parents).

*Notes:* Twins fixed effects models. Standard errors are in parentheses.

*** = 0.001, ** = 0.01, * = 0.05.

Controls: gender of the twin.

### Sensitivity analyses

I run several additional analyses to increase the reliability of the main results.

First, I introduce a control for early cognitive ability. Given that previous literature has pointed out that parents respond to the levels of cognitive skills of their children, I want to dismiss the possibility of health problems just being a signal of lower cognitive skills for parents. These results are presented in Supplementary Appendix Table A3 and show that the main estimates remain the same, with some small variations in their size.

The second sensitivity check implemented aims to evaluate the effect of birth weight on parental responses since it is the most common alternative measure to the twin medical risk composite. Supplementary Appendix Table A4 shows that, although the twin with a larger birth weight receives less negative parental responses at age 3, these effects are substantially smaller and stop being significant at ages 4 and 7. Supplementary Appendix Table A5 shows the results when both the Twin Medical Risk and birth weight are introduced at the same time in the models. In this scenario, birth weight remains mostly insignificant, and the twin medical scale maintains its previous effect.^5^

Given the relevance of gender in all the models, I rerun the results, including a gender interaction effect (Supplementary Appendix Table A6). Despite male twins are, in general, more likely to receive negative parental responses, there are no differences in how parents respond to health problems regarding the gender of the twin. Similarly, Supplementary Appendix Table A7 shows the main results when accounting for the specific parent (mother or father) answering the questionnaires about twins’ health and parental responses. The results stay unaffected.

Since the zygosity of the twins has been shown to affect parental responses in previous literature (Asbury *et al.*, 2003; Asbury, Dunn and Plomin, 2006; Viding *et al.*, 2009), in Supplementary Appendix Table A8 I present the main results with an interaction term for monozygotic twins, who are 33 per cent of the whole dataset. On average, there do not seem to exist significant differences in how parents respond emotionally or in terms of discipline behaviours regarding the zygosity of the twins.

Finally, I dig deeper into the heterogeneous SES effects on parental responses and rerun the models using (i) an extended operationalization of parental education with six categories (Supplementary Appendix Table A9), (ii) a variable measuring maternal occupation (Supplementary Appendix Table A10), and (iii) household income (Supplementary Appendix Table A11). In all three cases, the results are in line with the estimates found for the reduced operationalization of parental education. All this evidence suggests there are generally no differences by SES in how parents respond to the health disadvantages of their children. Moreover, I rerun the interaction model between health and parental socioeconomic status in Supplementary Appendix Table A12 exploiting between-families differences. Although these OLS results cannot be interpreted in causal terms, it is important to notice that they are consistent with the fixed-effects ones presented in Table 2.

An alternative explanation for these null results in the stratified analyses is that some of the parental responses analyzed in this article are more socially classed than other. This would be the case if, for instance, certain behaviours were very common among high-SES families. In that case, it could be that parents were not modifying their parental behaviour as a response to their children’s health simply because it is a very central part of their parenting and, therefore, quite inelastic to children’s characteristics^6^ (Bernardi and Triventi, 2020; Breinholt and Conley, 2023). Previous literature has suggested that high-SES parents are especially likely to explain things to their children (Hart and Risley, 1995), as opposed to the more common use of physical discipline that low-SES families do (Lareau, 2011). To account for this possibility, Supplementary Appendix Table A13 replicates the stratified analyses for each of the sub-items that form the parental discipline scale. Results shows that there are not relevant differences by group despite considering individually each of the discipline items.

## Discussion

Experiencing an early health problem crucially impacts a child’s life and prospects (Almond and Currie, 2011). But the role that parental responses play in alleviating or aggravating the long-term consequences of early health problems remains unclear (Rosales-Rueda, 2014; Yi *et al.*, 2015). Using rich TEDS data that follow twins over their childhood and young adulthood and implementing a series of within-twins fixed effects models, this article aims to answer two questions that shed light on this issue.

First, how do parents respond to the early health disadvantages suffered by their children? The empirical evidence offered here suggests that parents develop more negative emotional responses to the twin who has had a worse health status during their first 18 months of life. This is constant both in a within-family (twins fixed effects) and between-family (OLS models) context, as well as across the whole childhood period (ages 3, 4, and 7). Likewise, parents also implement harsher discipline behaviours with the sick twin, as compared to the healthier one. In both cases, this evidence suggests that parents are reinforcing early disadvantages by implementing less positive parental strategies when their children experience health problems.

Regarding the effects sizes, in a family in which a twin presents a risk score of −1 (better health) and the other twin of 1 (slightly worse health), the difference in parental responses would be around 0.122, which is one-tenth of a standard deviation of the outcome variable. This effect size is not negligible, especially when found in the context of an intra-family estimation. Moreover, the magnitude of the effect increases over time, which suggests that parental responses could get more important in later phases of children’s life cycles. These results go in line with Rosales-Rueda’s (2014) exploration of parental responses to mental health problems.

The second question of this article addresses whether parents from high- and low-SES respond differently to their children’s early health problems. The results in this sense go against what I expected: parents from more and less advantaged backgrounds respond to their children’s health disadvantages similarly. This is true regardless of the chosen SES operationalization (i.e., parental education, occupation, or income). An explanation for these results might be that, whereas previous literature has examined direct monetary investments, I am focusing on parental emotional and discipline responses, which are less resource-dependent. Although surprising, these results are coherent with previous studies such as Grätz and Torche’s (2016), which do not find stratification in parental responses to birth weight.

The relevance of these results is driven by the fact that, from a life-course perspective, the disadvantages experienced during the early childhood period will accumulate and eventually trigger further negative consequences for children’s adult lives (DiPrete and Eirich, 2006). If, on top of this, parental emotional and discipline responses deepen this negative impact on children’s later-in-life outcomes (Currie, 2009), the future prospects for the children can get seriously affected. This seems to be the case according to the existing literature showing that parental emotional and discipline responses negatively impact children’s developmental outcomes (Mullineaux *et al.*, 2009; Wang and Kenny, 2014), and speaks to the relevance that early childhood parental practices have in determining later-in-life inequalities.

This article makes four contributions to the literature. First, it examines how two previously overlooked types of parental responses, namely, emotional responses and discipline behaviours are affected by early health problems. Second, it does so by using a twin longitudinal dataset from the United Kingdom, where all the population has access to a comprehensive public health system. Third, the TEDS data have allowed me to consider repeated measures of parental responses over childhood, which opens the door to examining different rates at which parental responses are shaped as well as long-term outcomes. Finally, this article adds to the previous literature exploring parental responses to health disadvantages by considering heterogeneous SES effects, which has been noted to be a crucial dimension to understanding parental responses to other types of early child disadvantages.

This study has also some limitations. First, similarly to most of the literature studying parental responses with observational data, this article does not observe which is the exact factor that triggers the response. Although the sensitivity test accounting for early cognitive abilities suggests that parents are not interpreting health problems as pure cognitive skills measures, there is still a possibility that parental responses are originated as a response to unobserved factors.

Second, it is likely that when parents develop negative responses to their child’s health problems, this has spillovers into the other twin, whose home environment will also be affected by the stressful event. In this case, and given that the twin fixed-effects model exploits differences within the twins, the obtained estimates would be underestimating the effect of children’s health disadvantages on parental responses. However, this also means that the exact magnitude of the effects found in this article must be interpreted with caution.

Related to the last point, it could be the case that spillovers of parental emotions or behaviours are more common between twins than between other siblings, which would compromise the external validity and generalizability of these results (Abufhele, Behrman and Bravo, 2017). A positive note in this sense is that the between-OLS models estimated also show that health problems trigger more negative responses, which suggests that this relationship operates not only on an intra-family but also an inter-family level. Moreover, from a theoretical perspective, one would expect parents to find it harder to detect differences between twins than between other siblings. Therefore,this also suggests that the estimates presented here would underestimate the effect that early health problems have on parental responses.

Future research should also explore whether parental responses are sensitive to different gradients of severity in health disadvantages by exploring parental responses to more extreme health problems, which are omitted from this study.

Policies targeted to improve the living conditions and prospects of ill children could benefit from implementing a more comprehensive family approach. For instance, policy interventions designed to help parents apply more cognitively stimulating parenting have shown beneficial results on children’s cognitive development. Similarly, public interventions could provide parents with more information on how they should respond to their children experiencing a health disadvantage to reduce its long-term impact on the children’s lives.

## Supplementary Material

jcae016_suppl_Supplementary_Material

## Data Availability

TEDS data is available upon request and approval from the pertinent organism. The TEDS data access policy can be found here: https://www.teds.ac.uk/researchers/teds-data-access-policy.

## Footnotes

1. The single-factor solution presented an eigenvalue of 2.62 and accounted for 65.67 per cent and 65.68 per cent of the variance for the first and second-born twins, respectively (Pike *et al.*, 2006, p. 58).

2. Note that the author is reproducing here the process of construction of the composites as described by the TEDS data centre, but the dataset provided already contains the final variable, so the author has not personally implemented these steps.

3. The ICC measures the level of similarity between individuals from the same group—in this case, twins nested into families—for a specific trait. Higher coefficients capture more similarity within the family.

4. Under normal circumstances, characteristics that do not vary between the twins, such as parental socioeconomic status, could not be part of the within-twins fixed effects models. However, I am using the exception contemplated by Allison (2009) in his seminal work about fixed effects to test these moderation effects: an invariant variable might be introduced if and only if it interacts with a variant variable (in this case, one which varies between twins, such as early health problems).

5. This piece of results should be interpreted with caution given the very likely collinearity issues present in the model.

6. The author is grateful to an anonymous reviewer for this suggestion.

## Notes

Alicia García-Sierra is a Postdoctoral Researcher at the Swiss Centre of Expertise in Life Course Research LIVES at the University of Lausanne working on the EQUALOPP project. She obtained her PhD from the University of Oxford and Nuffield College in January 2024, supported by a Clarendon Scholarship.
