# Supplementary material for: Parental responses to children’s early health disadvantages: evidence from a British twin study
Source: Eur Sociol Rev. 2024 Mar 18;41(1):97–110. doi: 10.1093/esr/jcae016 (PMC11979777; doi:10.1093/esr/jcae016)
Supplement: jcae016_suppl_Supplementary_Material [file jcae016_suppl_supplementary_material.pdf]

SUPPLEMENTARY MATERIALS

Parental responses to children's early health disadvantages: evidence from a British twin study.

Alicia García-Sierra

Appendix A - Tables

Table A1. Descriptive Statistics.

Variable	*Mean/ Proportion*	*SD*	*Type of Variable*	*Min*	*Max*	*Measured at Age*	*Original Variable*	*Reported by Parents (P) or Twins (T)*
Twin Medical Risk Scale	0	1	Continuous	-3	6	1	*atwmed1*	P
Emotional Age 3	0	1	Continuous	-6	6	3	*cbfac11*	P
Emotional Age 4	0	1	Continuous	-6	6	4	*dbfac11*	P
Emotional Age 7	0	1	Continuous	-6	6	7	*gbfac11*	P
Discipline Age 3	0	1	Continuous	-6	6	3	*cdisto1*	P
Discipline Age 4	0	1	Continuous	-6	6	4	*ddisto1*	P
Discipline Age 7	0	1	Continuous	-6	6	7	*gdisp1*	P
Educational Performance	8.89	1.2	Continuous	4	11	16	*pcexgcsecorem1*	T
Subjective-Health	2.53	1.01	Continuous	1	5	21	*u1crand11*	T
Parental Education	Tertiary-Educated 27%	-	Binary	0	1	1	*amohqual afahqual*	P
Twin's Gender	Male 49,9%	-	Binary	0	1	1	*sex1*	P
Age of the mother at birth	30.73	4.84	Continuous	16	45	-	*amumagetw*	P
Number of siblings	0.04	0.22	Continuous	0	6	7	*anyngsib*	P
Birth Weight (g)	2476.1	559	Continuous	397.2	4560	1	*arkidgr1*	P
Cognitive Ability at Age 3	0	1	Continuous	-3	3	3	*cparca1*	P
Zygosity	Monozyg 33%	-	Binary	0	1	1	*zygos*	P
Maternal Occupation	Service Class 50.1%	-	Binary	0	1	1	*ppmoemplc*	P

Gender of the Parent-Respondent	Father 1.57%	-	Binary	0	1	1	*aad1rel*	P
Household Income	7.89	2.94	Categorical	1	11	1	*pphhincc*	P

Table A2. Parental Responses. OLS Models.

	Dependent Variable					
	**Negative Emotional Responses**			**Negative Discipline Behaviours**		
	Age 3	Age 4	Age 7	Age 3	Age 4	Age 7
	(1)	(2)	(3)	(4)	(5)	(6)
Twin Medical Risk Scale	0.039***	0.044***	0.034***	-0.001	0.016*	0.033***
	(0.010)	(0.009)	(0.009)	(0.010)	(0.009)	(0.009)
Male	0.126***	0.139***	0.130***	0.130***	0.185***	0.266***
	(0.020)	(0.017)	(0.017)	(0.019)	(0.017)	(0.017)
Tertiary-Educated	0.058**	0.057**	0.094***	-0.269***	-0.242***	-0.090***
	(0.022)	(0.019)	(0.019)	(0.022)	(0.019)	(0.019)
Mother's Age at Birth	0.003	0.002	-0.0001	-0.017***	-0.008***	-0.008***
	(0.002)	(0.002)	0.002)	(0.002)	(0.002)	(0.002)
Number of Siblings	-0.012	-0.060	-0.019	0.095	0.128**	0.158***
	(0.058)	(0.044)	(0.044)	(0.056)	(0.043)	(0.044)
Constant	-0.168*	-0.125*	-0.090	0.555***	0.243***	0.129*
	(0.070)	(0.061)	(0.062)	(0.069)	(0.061)	(0.061)
Observations	10,154	13,542	13,066	10,154	13,542	13,066
R^2	0.006	0.008	0.007	0.032	0.026	0.024

Note: Standard Errors are in parentheses. ****=0.001, **=0.01, *=0.05.*

Table A3. Parental Responses and Cognitive Ability Control

	Dependent Variable					
	**Negative Emotional Responses**			**Negative Discipline Behaviours**		
	Age 3 (1)	Age 4 (2)	Age 7 (3)	Age 3 (4)	Age 4 (5)	Age 7 (6)
Twin Medical Risk Scale	0.051* (0.022)	0.054** (0.018)	0.054* (0.021)	-0.016 (0.020)	0.032* (0.015)	0.022* (0.01)
Cognitive Ability	-0.082*** (0.018)	-0.032* (0.015)	-0.086*** (0.014)	-0.021 (0.012)	-0.070*** (0.018)	-0.041* (0.018)
Male	0.144*** (0.023)	0.264*** (0.020)	0.132*** (0.018)	0.236*** (0.016)	0.109*** (0.022)	0.293*** (0.023)
Observations	9,457	15,196	11,781	9,457	15,196	11,781

Note: Standard Errors are in parentheses. ****=0.001, **=0.01, *=0.05.*

Table A4. Parental Responses to Birth Weight

	Dependent Variable					
	**Negative Emotional Responses**			**Negative Discipline Behaviours**		
	Age 3 (1)	Age 4 (2)	Age 7 (3)	Age 3 (4)	Age 4 (5)	Age 7 (6)
Birth weight	-0.0001* (0.00003)	-0.00002 (0.00002)	-0.00001 (0.00002)	-0.0001** (0.00002)	-0.00001 (0.00003)	-0.00001 (0.00003)
Male	0.158*** (0.020)	0.160*** (0.018)	0.155*** (0.019)	0.259*** (0.018)	0.244*** (0.016)	0.315*** (0.020)
Observations	11,640	15,463	15,026	11,640	15,463	15,026

Note: Standard Errors are in parentheses. ****=0.001, **=0.01, *=0.05.*

Table A5. Parental Responses and Birth Weight Control

	Dependent Variable					
	**Negative Emotional Responses**			**Negative Discipline Behaviours**		
	Age 3 (1)	Age 4 (2)	Age 7 (3)	Age 3 (4)	Age 4 (5)	Age 7 (6)
Twin Medical Risk Scale	0.052* (0.025)	0.048* (0.022)	0.050* (0.024)	-0.019 (0.022)	0.032* (0.015)	0.049* (0.024)
Birth Weight	-0.00001 (0.00003)	-0.00004 (0.00003)	-0.00004 (0.00003)	-0.00003 (0.00003)	-0.0001* (0.00003)	-0.0001 (0.00003)
Male	0.162*** (0.021)	0.162*** (0.018)	0.154*** (0.020)	0.252*** (0.018)	0.248*** (0.016)	0.317*** (0.020)
Observations	11,273	14,903	14,404	11,273	14,903	14,404

Note: Standard Errors are in parentheses. ****=0.001, **=0.01, *=0.05.*

Table A6. Parental Responses and Gender Interaction

	Dependent Variable					
	**Negative Emotional Responses**			**Negative Discipline Behaviours**		
	Age 3 (1)	Age 4 (2)	Age 7 (3)	Age 3 (4)	Age 4 (5)	Age 7 (6)
Twin Medical Risk Scale (ref. categ: female)	0.051* (0.023)	0.072*** (0.020)	0.056** (0.021)	-0.025 (0.020)	0.036* (0.017)	0.048* (0.022)
Male	0.160*** (0.020)	0.154*** (0.018)	0.149*** (0.019)	0.251*** (0.018)	0.240*** (0.015)	0.310*** (0.020)
Medical Risk x Male	-0.010 (0.021)	-0.021 (0.017)	-0.002 (0.019)	0.027 (0.018)	-0.004 (0.015)	-0.012 (0.019)
Observations	11,525	15,295	14,745	11,525	15,295	14,745

Note: Standard Errors are in parentheses. ****=0.001, **=0.01, *=0.05.*

Table A7. Parental Responses and Parental Gender Control

	Dependent Variable					
	**Negative Emotional Responses**			**Negative Discipline Behaviours**		
	Age 3<br>(1)	Age 4<br>(2)	Age 7<br>(3)	Age 3<br>(4)	Age 4<br>(5)	Age 7<br>(6)
Twin Medical Risk Scale<br>(ref. categ: mother answering)	0.046*<br>(0.020)	0.062***<br>(0.018)	0.055**<br>(0.019)	-0.010<br>(0.018)	0.062***<br>(0.018)	0.042*<br>(0.019)
Medical Scale x Father Answering	0.014<br>(0.186)	-0.102<br>(0.214)	0.041<br>(0.203)	-0.053<br>(0.163)	-0.102<br>(0.214)	-0.023<br>(0.204)
Observations	11,525	15,295	14,745	11,525	15,295	14,745

Note: Standard Errors are in parentheses. ****=0.001, **=0.01, *=0.05.*

Table A8. Parental Responses and Zygosity Interaction

	Dependent Variable					
	**Negative Emotional Responses**			**Negative Discipline Behaviours**		
	Age 3<br>(1)	Age 4<br>(2)	Age 7<br>(3)	Age 3<br>(4)	Age 4<br>(5)	Age 7<br>(6)
Twin Medical Risk Scale<br>(ref. categ: dizyg)	0.025<br>(0.024)	0.061***<br>(0.021)	0.041*<br>(0.023)	-0.030<br>(0.021)	0.028<br>(0.019)	0.013<br>(0.024)
Medical Scale x Monozygotic	0.040<br>(0.044)	-0.022<br>(0.039)	0.023<br>(0.041)	0.018<br>(0.039)	-0.019<br>(0.034)	0.042<br>(0.042)
Observations	11,525	15,295	14,745	11,525	15,295	14,745

Note: Standard Errors are in parentheses. ****=0.001, **=0.01, *=0.05.*

Table A9. Parental Responses by Extended Parental Education

	Dependent Variable					
	**Negative Emotional Responses**			**Negative Discipline Behaviours**		
	Age 3<br>(1)	Age 4<br>(2)	Age 7<br>(3)	Age 3<br>(4)	Age 4<br>(5)	Age 7<br>(6)
Twin Medical Risk Scale (ref. category: No-Education)	-0.054<br>(0.102)	-0.094<br>(0.109)	-0.081<br>(0.107)	0.021<br>(0.087)	0.022<br>(0.096)	-0.105<br>(0.108)
Medical Risk x Secondary-Education	0.070<br>(0.108)	0.153<br>(0.113)	0.169<br>(0.112)	-0.057<br>(0.093)	-0.007<br>(0.100)	0.188<br>(0.113)
Medical Risk x Vocational	0.116<br>(0.116)	0.202<br>(0.120)	0.143<br>(0.120)	-0.003<br>(0.100)	-0.014<br>(0.106)	0.190<br>(0.121)
Medical Risk x A-levels	0.024<br>(0.118)	0.108<br>(0.120)	0.101<br>(0.119)	-0.102<br>(0.102)	-0.052<br>(0.106)	0.138<br>(0.120)
Medical Risk x Undergraduate Studies	0.206<br>(0.114)	0.190<br>(0.117)	0.173<br>(0.116)	0.0001<br>(0.098)	0.106<br>(0.104)	0.116<br>(0.117)
Medical Risk x Postgraduate Studies	0.133<br>(0.118)	0.198<br>(0.119)	0.044<br>(0.120)	-0.015<br>(0.101)	0.012<br>(0.105)	0.058<br>(0.121)
Controls	Yes	Yes	Yes	Yes	Yes	Yes
Observations	11,525	15,295	14,745	11,525	15,295	14,745

Note: Standard Errors are in parentheses. ****=0.001, **=0.01, *=0.05*. The extended parental education variable follows the same logic of construction as the dichotomous version used in the main analysis, ie., a dominance model, but without clustering the categories into two only groups.

Table A10. Parental Responses by Maternal Occupation

	Dependent Variable					
	**Negative Emotional Responses**			**Negative Discipline Behaviours**		
	Age 3 (1)	Age 4 (2)	Age 7 (3)	Age 3 (4)	Age 4 (5)	Age 7 (6)
Twin Medical Risk Scale (ref. category: Working Class)	0.013 (0.026)	0.0003 (0.050)	0.002 (0.050)	-0.063 (0.042)	-0.020 (0.040)	0.004 (0.051)
Twin Medical Risk x Service Class	0.107 (0.066)	0.103 (0.071)	0.041 (0.071)	0.101* (0.060)	0.028 (0.057)	-0.051 (0.073)
Controls	Yes	Yes	Yes	Yes	Yes	Yes
Observations	4,364	4,142	4,294	4,364	4,142	4,294

Note: Standard Errors are in parentheses. ****=0.001, **=0.01, *=0.05.* Following the reduced Goldthorpe scheme, the "service class" includes those individuals in professional and managerial groups, and the "working class" is those skilled and unskilled manual workers.

Table A11. Parental Responses by Household Income

	Dependent Variable					
	**Negative Emotional Responses**			**Negative Discipline Behaviours**		
	Age 3 (1)	Age 4 (2)	Age 7 (3)	Age 3 (4)	Age 4 (5)	Age 7 (6)
Twin Medical Risk x Household Income	0.006* (0.004)	0.0003 (0.003)	0.005 (0.004)	0.001 (0.003)	0.001 (0.004)	-0.003 (0.004)
Controls	Yes	Yes	Yes	Yes	Yes	Yes
Observations	4,364	4,142	4,294	4,364	4,142	4,294

Note: Standard Errors are in parentheses. ****=0.001, **=0.01, *=0.05.*
This variable Household Income Level presents 11 categories, although I am treating it as continuous one in the analyses for simplification. 1=Under £5,000, 2=£5,000 to £9,999, 3=£10,000 to £14,999, 4=£15,000 to £19,999, 5=£20,000 to £24,999, 6=£25,000 to £29,999, 7=£30,000 to £39,999, 8=£40,000 to £49,999, 9=£50,000 to £74,999, 10=£75,000 to £100,000, 11=Over £100,000.

Table A12. Parental Responses. OLS Models with interaction.

	Dependent Variable					
	**Negative Emotional Responses**			**Negative Discipline Behaviours**		
	Age 3	Age 4	Age 7	Age 3	Age 4	Age 7
	(1)	(2)	(3)	(4)	(5)	(6)
Twin Medical Risk Scale	0.034** (0.012)	0.044*** (0.009)	0.034*** (0.009)	-0.001 (0.012)	0.016 (0.01)	0.032*** (0.01)
Tertiary-Educated	0.059** (0.022)	0.057** (0.019)	0.094*** (0.019)	-0.269*** (0.022)	-0.242*** (0.019)	-0.090*** (0.019)
Medical Risk * Tertiary Educated	0.016 (0.022)	-0.025 (0.018)	-0.016 (0.019)	0.002 (0.022)	-0.0002 (0.018)	0.001 (0.019)
Constant	-0.169* (0.070)	-0.123* (0.061)	-0.090 (0.062)	0.555*** (0.069)	0.243*** (0.061)	0.129* (0.061)
Observations	10,154	13,542	13,066	10,154	13,542	13,066
R^2	0.006	0.008	0.007	0.032	0.026	0.024

Note: Standard Errors are in parentheses. ****=0.001, **=0.01, *=0.05.* Controls include: gender, age of the mother and number of siblings.

Table A13. Subscales of the parental discipline index by SES

	Dependent Variable					
	**High SES**			**Low SES**		
	Smack and shout	Explain and being firm (reversed)	Make jokes and ask others to take responsibility	Smack and shout	Explain and being firm (reversed)	Make jokes and ask others to take responsibility
	(1)	(2)	(3)	(4)	(5)	(6)
Twin Medical Risk Scale	0.026 (0.032)	-0.005 (0.025)	-0.005 (0.025)	-0.034 (0.022)	-0.012 (0.016)	-0.012 (0.016)
Observations	3,043	3,053	3,053	7,146	7,141	7,141

Note: Standard Errors are in parentheses. ****=0.001, **=0.01, *=0.05.* Control for gender included.

Appendix B – Potential Biases

There are several biases and sources of measurement error that could be playing a role in these models. Some of them are common to all the within-family estimators, whereas others are specifically related to the nature of the independent or dependent variables used in this paper.

The first one is the potential reporting bias, which would imply that some parents are more likely to report health problems given certain observed or unobserved characteristics (Yi et al., 2015). Within-twins estimators solve this problem since I am looking at reports made by the same parent for each of their children. It is still a possibility that reporting biases differ by SES, which would be influential for the third part of the results presented in this study. However, an exploration of the mean values of health risks for each group (high- and low-SES) shows that there are no significant differences in the reported health status of the children.

A specific type of this reporting bias can be considered the social desirability bias, or the tendency to answer surveys with what is expected to be a socially correct answer. Since parents do not want their children to be ill, they could tend to misreport health problems. To deal with this aspect, the twin medical risk composite includes objective measures of health such as days stayed in the hospital or time spent in special care. This would also partially account for the possibility of parents reporting more health problems only when the child is very ill, but not when the child is only mildly sick (i.e., reporting bias by responses).

The second type of bias to consider is the attenuation bias, generated by a measurement error problem in the independent variables and that would bias the estimates towards zero. This is especially relevant in this study for two reasons. First, as Griliches (1979) shows, the within-twin estimator can exacerbate the problem of measurement error. Second, as shown by Strauss & Thomas (1998), self-reported health is particularly prone to incorporate measurement error. However, I suggest that there are several advantages of the data and methods used in this paper that minimize this problem.

First, it is important to notice that I am not using self-reported health measures, but parental-reported ones. These are collected by the interviewers who ask the parents about the health

status of their children through very concrete questions (i.e., "how many days has the twin been in special care in the last year"?) to which parents have to offer accurate answers (i.e., the exact number of days). This should partially alleviate Strauss & Thomas' (1998) concern about self-reported measures.

On top of this, another potential source of measurement error is the one arriving from different respondents having different thresholds to consider a health condition a problem. But, as Yi et al. (2015) state, this is overcome by the twin design again, since parents would implement the same threshold with the two children. Besides, there is a danger of measurement errors coming from recalling bias (i.e., parents whose child is currently ill might be more likely to report that the child has been ill in the past). However, following Yi et al. (2015) on this point too, given the young age of the twins (maximum 18 months) it is unlikely that recall bias could play a role in the results.

Regarding the presence of measurement error in the second part of the analysis, when parental responses are used as independent variables, the risk is that parents find it difficult to notice differences in treatment between their children, especially when they are monozygotic twins (Abufhele et al., 2017). However, the way these questions are formulated in the TEDS survey, emphasizing the difference in treatment between the twins, ensures a certain level of variation within the family. Thus, I argue that the risk of the results presented here being attenuated by the presence of measurement error is unlikely. In addition, and as shown in the results section, the estimates are not biased toward zero.

A different potential bias to consider is selection bias. In sibling models, there is always the chance that parents make fertility decisions based on previous children's endowments, which would generate some selection problems. However, since twins share their birth date, this should not be a matter here (Ejrnæs and Pörtner, 2004; Ermisch and Francesconi, 2013; Grätz, 2015). Moreover, according to Savelyev et al. (2020, pp. 16), “as long as the omitted variable that affects sample selection is family-specific, the twin first differences model controls for it”.

Finally, the longitudinal design employed in this paper accounts for potential reverse causality problems. This is, if health aspects and parental responses were measured at the same time, it would be hard to assume that health problems are not affected by previous parental actions. However, given that health is measured in the very first stages of life here, and that parental

responses are captured between two and five years afterwards, this should circumvent the reverse causality issues.
